# Familial Alzheimer’s disease mutations in amyloid precursor protein impair calcineurin signaling to NMDA receptors

**DOI:** 10.1016/j.jbc.2024.108147

**Published:** 2024-12-26

**Authors:** Steven J. Tavalin

**Affiliations:** Department of Pharmacology, Addiction Science, and Toxicology, College of Medicine, The University of Tennessee Health Science Center, Memphis, USA

**Keywords:** Alzheimer’s disease, amyloid precursor protein, calcineurin, calcium, NMDA receptors, signal transduction, synaptic plasticity

## Abstract

Familial Alzheimer’s disease (FAD) is frequently associated with mutations in the amyloid precursor protein (APP), which are thought to lead to cognitive deficits by impairing NMDA receptor (NMDAR)-dependent forms of synaptic plasticity. Given the reliance of synaptic plasticity on NMDAR-mediated Ca^2+^ entry, shaping of NMDAR activity by APP and/or its disease-causing variants could provide a basis for understanding synaptic plasticity impairments associated with FAD. A region of APP (residues 639–644 within APP695) processed by the γ-secretase complex, which generates amyloid-β peptides, is a hotspot for FAD mutations. This region bears similarity to a binding motif for calcineurin (CaN), a Ca^2+^/calmodulin-dependent phosphatase. Interaction assays confirm that APP associates with CaN in native tissue as well as in a heterologous expression system. This capacity to bind CaN extends to APP family members amyloid precursor-like protein 1 and amyloid precursor-like protein 2 (APLP1 and APLP2, respectively). Electrophysiological analysis demonstrates that APP and its family members limit NMDAR activity, in a manner consistent with CaN-dependent regulation of NMDAR desensitization. FAD mutations, in this region of APP, impair this regulation and consequently enhance NMDAR activity. Thus, by altering the landscape for CaN regulation of NMDA receptors, FAD mutations in APP may contribute to faulty information processing by modifying the dynamic range and temporal window of a critical signal for synaptic plasticity.

NMDA receptors (NMDARs) act as synaptic coincidence detectors, contributing to many forms of activity-dependent synaptic plasticity thought to underlie episodic information acquisition, which represents an early aspect of cognitive function compromised in Alzheimer’s disease (AD) (1). NMDARs are heterotetrameric complexes, derived from combinations of three subunit families: GluN1, GluN2, and GluN3 (2). Each family member is further diversified by either alternative splicing (GluN1) or the presence of multiple isoforms (GluN2 and GluN3) (2). NMDARs assemble from dimers of heterodimers, with each heterodimer containing an obligate glycine-binding GluN1 subunit and either a glutamate-binding GluN2 or a glycine-binding GluN3 subunit, leading to numerous combinations (2). GluN2A subunit incorporation into NMDARs increases across development (2); thus, heteromeric assemblies of GluN1A/GluN2A are widely used as models for mature forms of NMDARs. NMDAR activity is shaped on a millisecond-to-second time scale by multiple forms of desensitization (3). Ca^2+^-dependent inactivation (CDI) and glycine-independent desensitization are feedback mechanisms that limit GluN2A-containing NMDAR activity (3, 4, 5). CDI arises from the rapid and reversible association of Ca^2+^/CaM with the GluN1 subunit (6, 7, 8, 9), while the extent of glycine-independent desensitization slowly increases *via* Ca^2+^/CaM-mediated CaN activation (3, 5), which is thought to modify GluN2A subunit phosphorylation (5). Both processes likely coexist under physiological conditions to ensure tight control of NMDAR receptor activity, as the current decline during agonist exposure remains substantial upon interference with either process (3, 10). Despite differences in subunit targets, these processes similarly impact the macroscopic time course of agonist-evoked currents and exhibit some mechanistic overlap at the single channel level (11, 12). Local Ca^2+^sources predominantly drive these processes as they are evident even in the presence of high concentrations of the fast Ca^2+^ chelator BAPTA (13, 14). While CaN activation ultimately impacts the amplitude and kinetics of synaptic NMDAR responses (12, 15, 16, 17), how CaN is targeted to NMDARs remains unclear.

Short linear motifs can serve as the principal interface for the recruitment of CaN to relevant substrates (18). The PxIxIT motif, the most well documented CaN short linear motif, was initially detected in the NFAT family of transcriptional regulators (19). Systematic substitution of amino acids within the core and adjacent positions of this motif led to the generation of the PVIVIT peptide, which can be used as an inhibitor of CaN targeting and activity within cells (19). Except for AKAP79/150 (20) and yeast Rcn1 (21), validated CaN-binding proteins that use the PxIxIT motif have the canonical proline. However, these deviations suggest that other residues may occupy this initial position and still enable CaN targeting. A region within APP (residues 639–644 in APP695, the predominant neuronal APP isoform), processed by the γ-secretase complex, matches the core PVIVIT peptide sequence, except for the presence of threonine in the initial position (Fig. 1, *A*–*C*). APP-family members APLP1 and APLP2 have conservative substitutions within their corresponding sequences compared to APP (Fig. 1*C*). While this region of APP is thought to reside within the APP transmembrane domain, the membrane spanning boundaries are debatable (22, 23) and nonhelical conformations of the transmembrane segment can be observed (24). Indeed, stretches of APP unwind to adopt a β strand conformation extending into the intracellular side of the membrane during initial γ-secretase cleavage (25). As the γ-secretase cleaves APP in a processive manner (26), it seems likely that a β strand conformation would not be limited to these residues. Thus, a β strand conformation of APP(639–644) could provide a compatible interface for CaN interaction as seen with PxIxIT motif-like peptides (27, 28). Collectively, this raised the prospect that the APP-family of proteins interacts with CaN *via* this PxIxIT motif variant. Moreover, as NMDARs interact with APP (29, 30, 31, 32) and are regulated by CaN (3, 5, 11, 12, 15, 16, 17), this opened the possibly that APP serves to recruit CaN to NMDARs.

**Figure 1:**
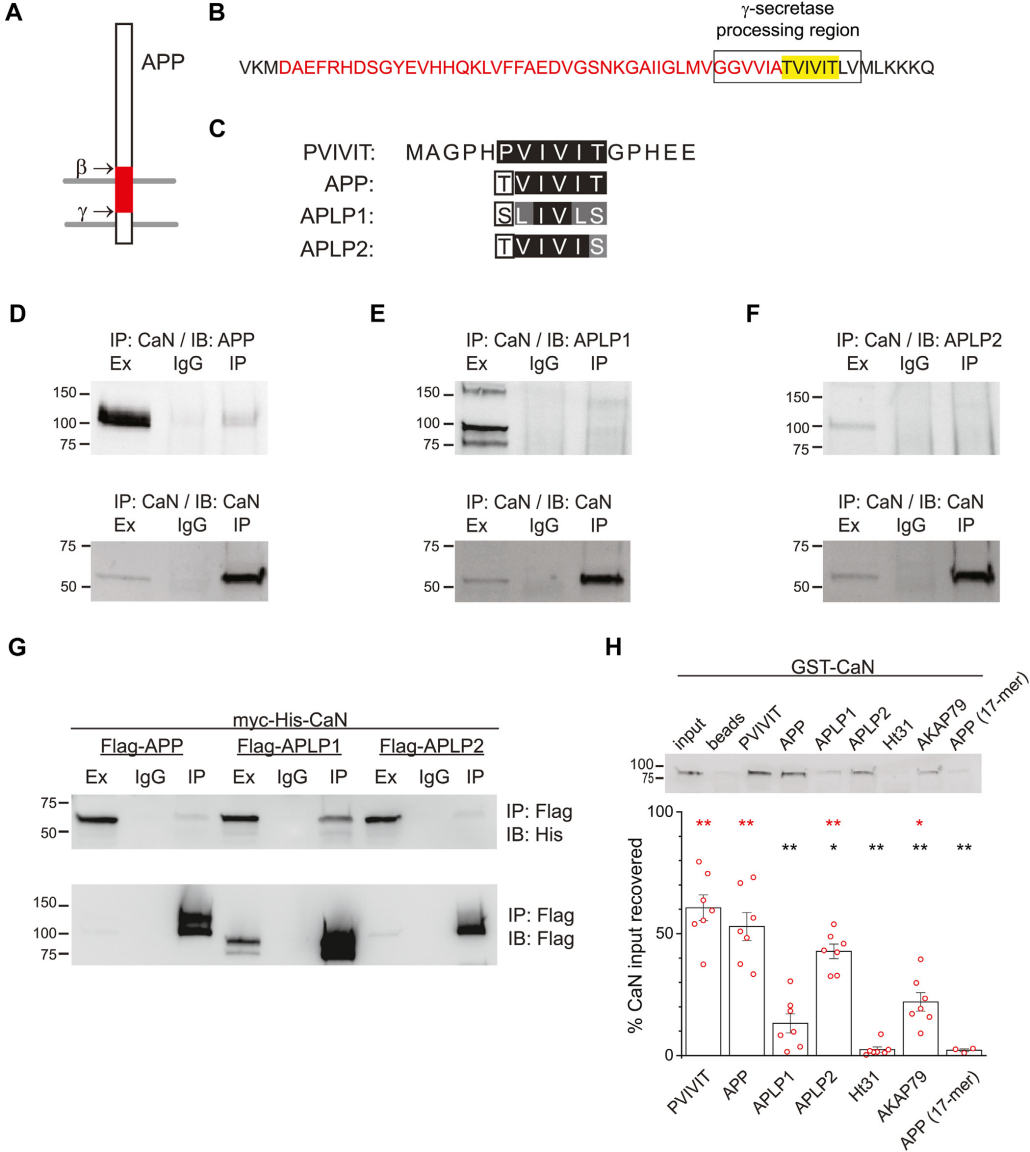
**APP family members bind calcineurin.***A*, schematic overview of the orientation of APP in the plasma membrane (*gray lines*) and the relative location of β- and γ-secretase cleavage sites yielding the Aβ peptide (*red*). *B*, expanded view of the region surrounding the Aβ42 (*red*) sequence of APP. The CaN-binding site is highlighted (*yellow*). *C*, amino acid sequence similarity between the PVIVIT motif peptide and the corresponding CaN-binding sites in APP family members (*black* background denotes identical; *gray* background denotes similar; *white* background denotes similarity with APP family members). *D*–*F*, representative western blots of co-IP experiments from rat brain extracts. Extracts (Ex) were subjected to immunoprecipitation (IP) with anti-CaN antibodies or a nonspecific IgG and immunoblotted (IB) for APP, APLP1, and APLP2 in panels *D*–*F*, respectively (*upper panels*). 5% of input was run in the extract lanes. Blots were stripped and reprobed with an anti-CaN antibody (*lower panels*). For validation of antibodies, see Fig. S1. *G*, representative co-IP experiments from HEK 293 cells transfected with myc-His-CaN and Flag-tagged versions of APP family members. Extracts were IP’d with anti-Flag antibodies and probed with anti-His antibodies (*upper panel*). Blots were stripped and reprobed with anti-Flag antibodies. *H*, *in vitro* interaction of recombinant GST-CaN (200 ng) with the indicated biotinylated peptides (10 μg). Peptides were precoupled to streptavidin-coated beads and incubated with GST-CaN (200 ng). Immunoblots were performed using anti-CaN antibodies. A representative blot is shown (*upper panel*). Summary bar graph from multiple experiments in shown (*lower panel*). Data are normalized to the GST-CaN standard (80 ng) and adjusted for the percentage of input represented by the standard (40%) and expressed as mean ± s.e.m. Overlaid individual data points are from biological replicates. Ht31 is a nonspecific control peptide, and beads represent a beads alone control. ∗*p* < 0.05; ∗∗*p* < 0.01 compared to PVIVIT (*black*) and Ht31 (*red*) evaluated by a one-way ANOVA (F_(6,38)_ = 32.050; *p* = 2.02 × 10^-13^ ∗∗) and Tukey’s *post hoc* test.

## Results

### APP family members can bind CaN

In support of this idea, APP reliably co-immunoprecipitated with CaN from rat brain homogenates obtained from 24–28-day-old rat pups (Fig. 1*D*). Some APLP1 species appeared to weakly associate with CaN (Fig. 1*E*), but the major APLP2 species could not be readily discerned (Fig. 1*F*). While APP family members exhibit similar developmental trajectories, peaking ∼1 to 4 weeks postnatally (33), their relative abundance to each other is not well-established. Thus, their abundance, along with their distinct subcellular dispositions (33), could determine their propensity to interact with CaN. Indeed, when expressed in HEK 293 cells, all flag-tagged APP family members were able to interact with myc-his-tagged CaN, except that APLP1 appeared to bind CaN most extensively (Fig. 1*G*). This may be due to its ability to achieve higher expression levels in this heterologous system (Fig. 1*G*). Due to these challenges in determining the relative affinity of APP family members for CaN, an *in vitro* binding assay was adopted, where the abundance of each could be controlled. A 17-amino acid peptide (APP-17-mer), based on the native APP sequence, exhibited minimal affinity for CaN, possibly due to the highly hydrophobic nature of this region and its tendency to self-aggregate (34). To circumvent this issue, the core CaN-binding sequence within the PVIVIT peptide was substituted with the corresponding sequence from each APP family member. This strategy has been used to evaluate the impact of sequence variation within the PxIxIT motif on CaN binding and provides a means to benchmark the relative affinity of this putative CaN-binding domain against its prototype as well as a CaN-binding peptide derived from AKAP79 (35). The peptide bearing the corresponding APP sequences bound CaN to nearly the same extent as the PVIVIT peptide (Fig. 1*H*). The APLP2-based peptide also exhibited considerable binding to CaN albeit it was ∼30% lower than the PVIVIT peptide (Fig. 1*H*). The APLP1-based peptide bound CaN to a more limited extent (∼80% lower than the PVIVIT peptide). Nonetheless, the APLP1-based peptide typically bound much greater than Ht31, a negative control peptide, and nearly as well as a peptide derived from the AKAP79 CaN-binding domain, which was included as an independent positive control (Fig. 1*H*). As the latter contributes to CaN-dependent signaling in cells (20), these data suggest that the APP family may, likewise, recruit CaN to relevant substrates.

### APP enables CaN-dependent regulation of NMDARs

As the principal calcium-dependent phosphatase in neurons, CaN couples activity-dependent Ca^2+^ signals to the phosphorylation state of a wide variety of synaptic substrates (36). NMDARs are not only subject to CaN-dependent regulation but also for critical for driving CaN activity linked to the weakening of AMPA receptor–mediated synaptic strength in the form of long-term depression, homeostatic synaptic downscaling, and by restricting long-term potentiation (36, 37). These forms of plasticity appear to be maladaptively engaged in AD and/or in response to amyloid-β (Aβ) (38, 39, 40). Immunocytochemical analysis indicates that APP exhibits partial overlap with the NMDAR GluN1 subunit (41) commensurate with the ability of APP family members to interact with NMDARs, possibly *via* the GluN1 subunit (29, 30, 31, 32). As APP interacts with CaN (Fig. 1) and NMDARs are subject to CaN-mediated regulation (3, 5, 11, 12, 15, 16, 17), NMDARs may represent a physiologically relevant target for APP-anchored CaN.

**Figure 2:**
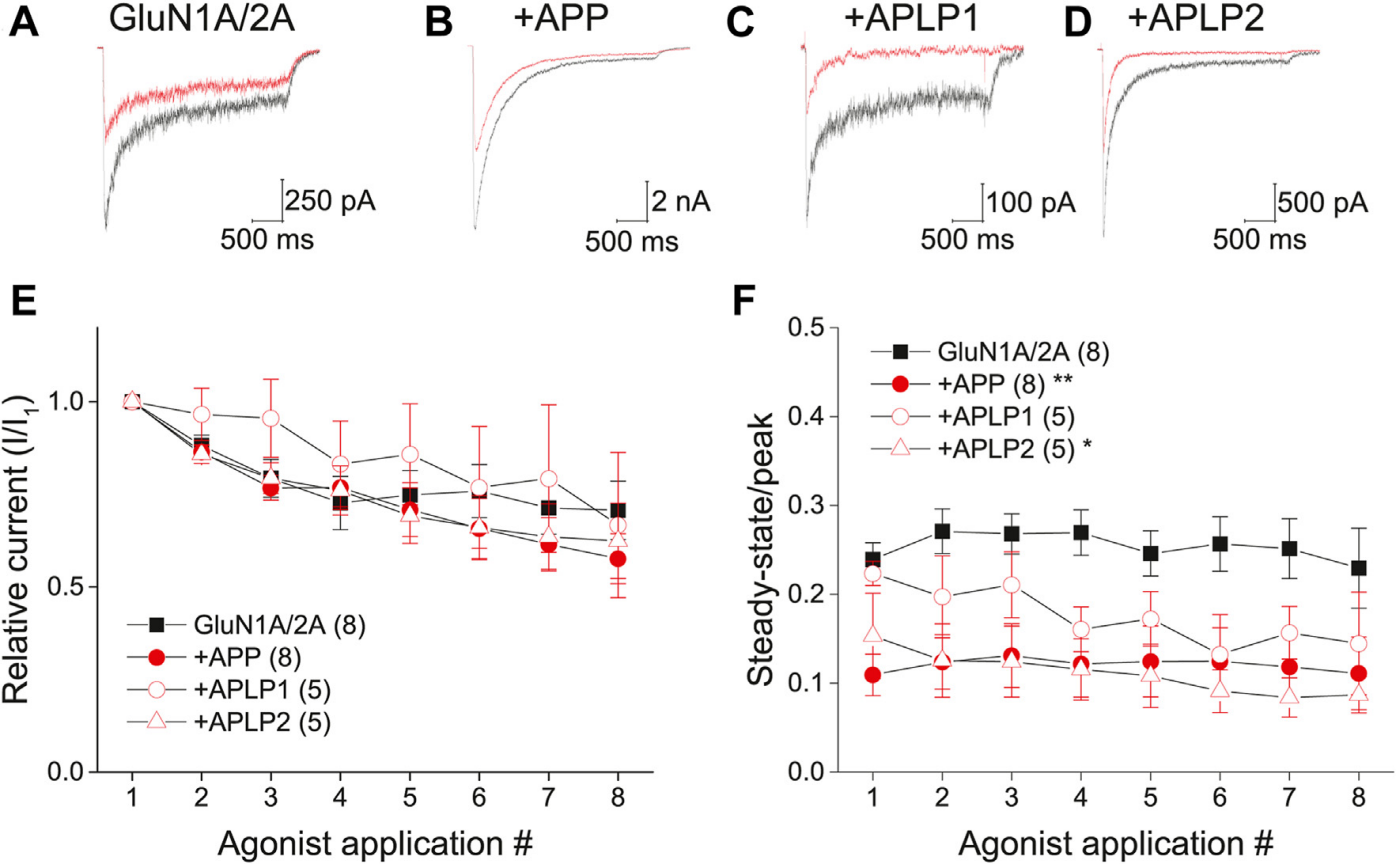
**APP family members control the extent of acute NMDAR desensitization.***A*–*D*, representative first (*black*) and last (*red*) NMDAR currents evoked by the application of 1 mM glutamate in the continuous presence of 100 μM glycine from HEK 293 cells expressing GluN1A/GluN2A receptor subunits and either GFP (*A*) or GFP-tagged versions of the indicated APP family members (*B–D*). Low concentrations of EGTA (0.2 mM) were included in the intracellular pipette solution. *E*, summary time course of the peak current response of each application normalized to the initial agonist application. *F*, summary time course of the extent of desensitization measured as the ss/p ratio for each agonist application. Data for (*E* and *F*) are represented as the mean ± s.e.m. The number of observations for each condition is indicated in parentheses and represents biological replicates. ∗*p* < 0.05; ∗∗*p* < 0.01 evaluated by repeated measures ANOVA (*E*: F_(3,22)_ = 32.050; *p* = 0.679; n.s.; *F*: F_(3,22)_ = 5.088; *p* = 0.008 ∗∗) followed by Dunnet’s *post hoc* test.

To test this idea, NMDAR currents were recorded from HEK 293 cells expressing GluN1A/GluN2A with either GFP or C-terminally GFP-tagged APP family members using a low intracellular concentration of the Ca^2+^ chelator EGTA (0.2 mM). Agonist stimulation protocols in which glycine-independent desensitization is expected to dominate the current decay during each agonist application were employed (3). Amplitudes of GluN1A/GluN2A currents declined by ∼ 30 to 40% (over the course of eight agonist applications delivered at 1/min) irrespective of the presence of APP family members (Fig. 2, *A*–*E*), reminiscent of a use-dependent process linked to tyrosine phosphatases (42). A progressive Ca^2+^/CaN-dependent increase in the desensitization of recombinant NMDARs, as measured by a reduction in the ratio of steady-state to peak (ss/p) current, which evolves within 10 min has been reported (3) and bears similarity to that in native tissue (5). In contrast, desensitization for GluN1A/GluN2A currents was stable across agonist applications (Fig. 2*F*), possibly due to the briefer agonist applications and/or the modestly higher EGTA concentration used here. Regardless, APP and APLP2 co-expression yielded NMDAR currents exhibiting stronger initial desensitization than GFP controls, which remained stable throughout the recording (Fig. 2, *B* and *D*, and *F*). Interestingly, APLP1 co-expression yielded currents with initial desensitization like that of GFP controls, but desensitization progressively increased to a level comparable to APP-expressing cells by the ∼ fourth glutamate application (Fig. 2, *C* and *F*). These effects are consistent with the idea that APP family members facilitate the functional coupling of CaN to the extent of NMDAR desensitization and appear to align well with their relative affinity to CaN. While GluN2B-containing NMDARs lack CDI under physiological conditions (4, 10), their desensitization is sensitive to Ca^2+^ buffering (43) and thus could also be subject to CaN-dependent regulation Indeed, APP co-expression also enhanced the extent to which GluN1A/GluN2B currents desensitized (Fig. S2). While the effect of APP on GluN2B-containing NMDARs was not as prominent as that for GluN2A-containing NMDARs, possibly due to the lower open probability associated with the GluN2B subunit (44, 45), these data are consistent with the ability of APP to interact with the most abundant NMDAR isoforms (29).

**Figure 3:**
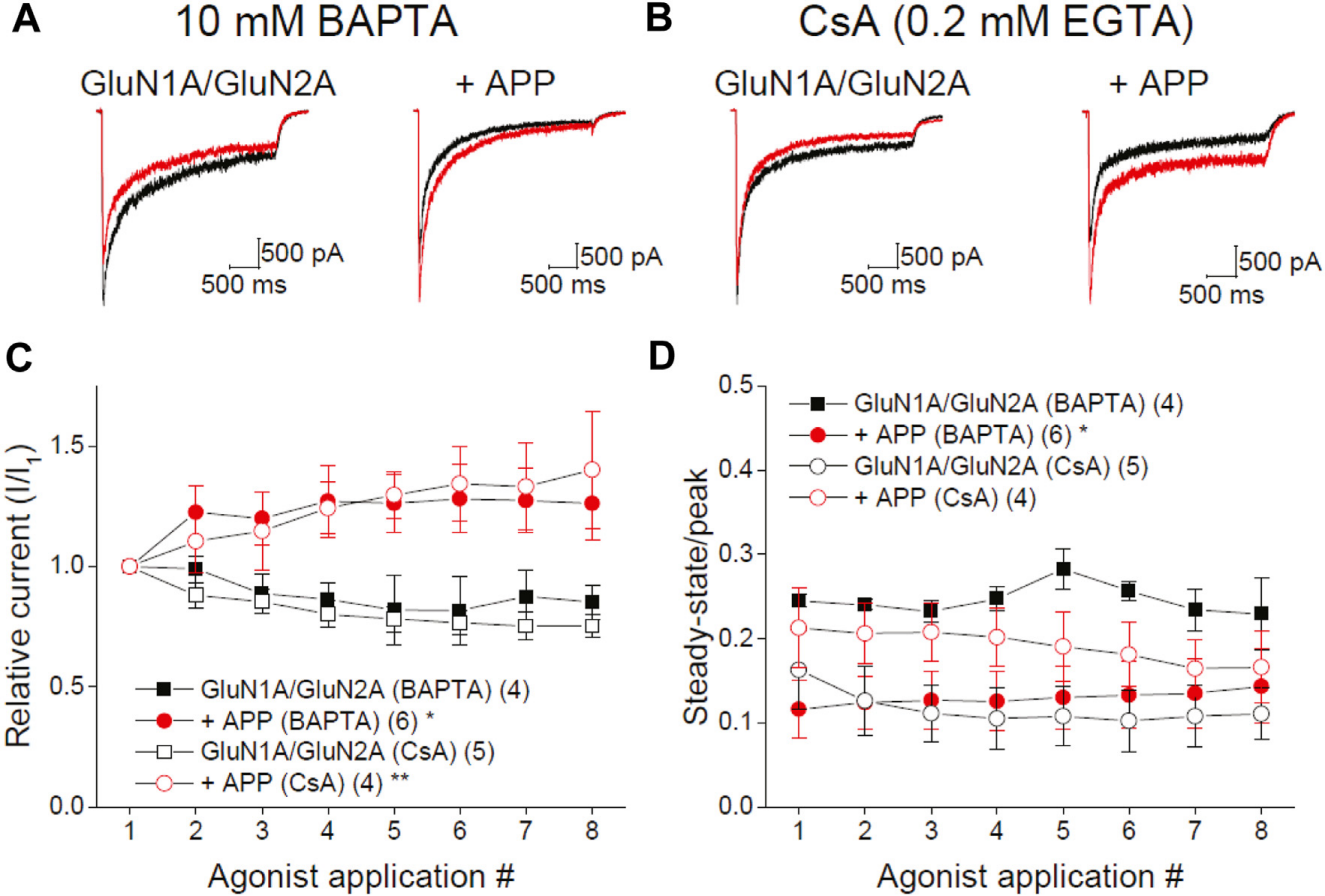
**APP controls NMDAR activity *via* CaN.***A* and *B*, representative first (*black*) and last (*red*) NMDAR currents evoked by the application of 1 mM glutamate in the continuous presence of 100 μM glycine from HEK 293 cells expressing GluN1A/GluN2A receptor subunits and either GFP or GFP-APP. 10 mM BAPTA was included in the intracellular pipette solution in panel (*A*). Cells were pretreated with CsA (10 min; 1 μM) and recorded in the continued presence of CsA using 0.2 mM EGTA in the intracellular pipette solution in panel (*B*). *C*, summary time course of the peak current response for each application normalized to the initial agonist application under conditions used in (*A* and *B*). *D*, summary time course of the extent of desensitization measured as the ss/p ratio for each agonist application under conditions used in (*A* and *B*). Data for (*C* and *D*) are represented as the mean ± s.e.m. The number of observations for each condition is indicated in parentheses and represents biological replicates. ∗*p* < 0.05; ∗∗*p* < 0.01 evaluated by repeated measures ANOVA (*C*: 10 mM BAPTA: F_(1,8)_ = 5.545; *p* = 0.046 ∗; CsA F_(1,7)_ = 13.339; *p* = 0.008 ∗∗; *D*: 10 mM BAPTA: F_(1,8)_ = 6.242; *p* = 0.037 ∗; CsA: F_(1,7)_ = 2.250; *p* = 0.177; n.s.) for APP compared to its respective control.

Inclusion of high concentrations of the fast Ca^2+^ chelator BAPTA in the intracellular recording solution can limit CaN-mediated decreases in NMDAR open probability and CaN-mediated increases in NMDAR desensitization (3, 5, 16). BAPTA (10 mM) caused a rapid (within 1–3 min) increase in the amplitude of GluN1A/GluN2A currents in APP-expressing cells, while GFP-expressing cells NMDAR currents declined in amplitude by a similar extent to that seen with low EGTA concentrations (Fig. 3, *A* and *C*; compare to Fig. 2*E*). This implies that APP controls the overall amplitude of the receptor by favoring a Ca^2+^-dependent process that limits NMDAR activity. Importantly, cells treated with a CaN inhibitor, cyclosporin A (CsA; 1 μM; 10 min), recorded with low intracellular EGTA, showed similar effects on NMDAR amplitude to that seen with high BAPTA concentrations (Fig. 3, *B* and *C*), strongly supporting the idea that APP-anchored CaN actively limits the availability of the NMDARs. BAPTA (10 mM), however, did not appear to modify the degree of NMDAR desensitization in GFP controls and APP-expressing cells, as this parameter was similar to that observed in low concentrations of intracellular EGTA (Fig. 3, *A* and *D*; compare to Fig. 2*F*). This lack of an effect of BAPTA could suggest that Ca^2+^ signals occurring very close to the NMDAR underlie the APP-induced increase in desensitization. The CaN inhibitor CsA, however, should be effective in blunting the effect of APP-anchored CaN irrespective of the location of the Ca^2+^ source. In register with this line of reasoning, the ss/p ratio in APP-expressing cells was no longer different (and modestly elevated) compared to GFP-expressing cells when CsA was present in recordings using low intracellular EGTA concentrations (Fig. 3*D*). It is interesting to note that in contrast to a previous study (3), desensitization appeared modestly enhanced in GFP-expressing cells treated with CsA relative to untreated cells (Fig. 3, *B* and *D*; compare with Fig. 2*F*). This difference may be related to the previous use of a GluN1 truncation mutant that lacks CDI (3) as opposed to the full-length GluN1 used here. Indeed, CaN activity has been associated with reductions in the effect of calmodulin on NMDARs (11), which contributes to CDI of NMDARs (9). Thus, inhibition of CaN with CsA may favor a shift towards the inactivation process when using the full-length GluN1A subunit. Nonetheless, these data indicate that NMDAR amplitude is enhanced, and NMDAR desensitization is reduced upon CaN inhibition when APP is co-expressed, further bolstering the idea that APP-anchored CaN limits NMDAR activity. Based on the differential sensitivity of these parameters to high concentrations of BAPTA, these data suggest that APP-linked CaN may be controlling two distinct processes. The first is the extent of desensitization during an agonist application, which could reflect the phosphorylation state of one or more sites within the vicinity of the channel. The second is the phosphorylation state of one or more sites, which controls the availability of NMDARs, and is resolved over repeated agonist exposures. This latter process may be more remotely localized than that controlling the ss/p ratio and may involve long-lived desensitization states (11) and/or trafficking of the receptor (29, 30).

### FAD mutations impair APP regulation of NMDARs

**Figure 4:**
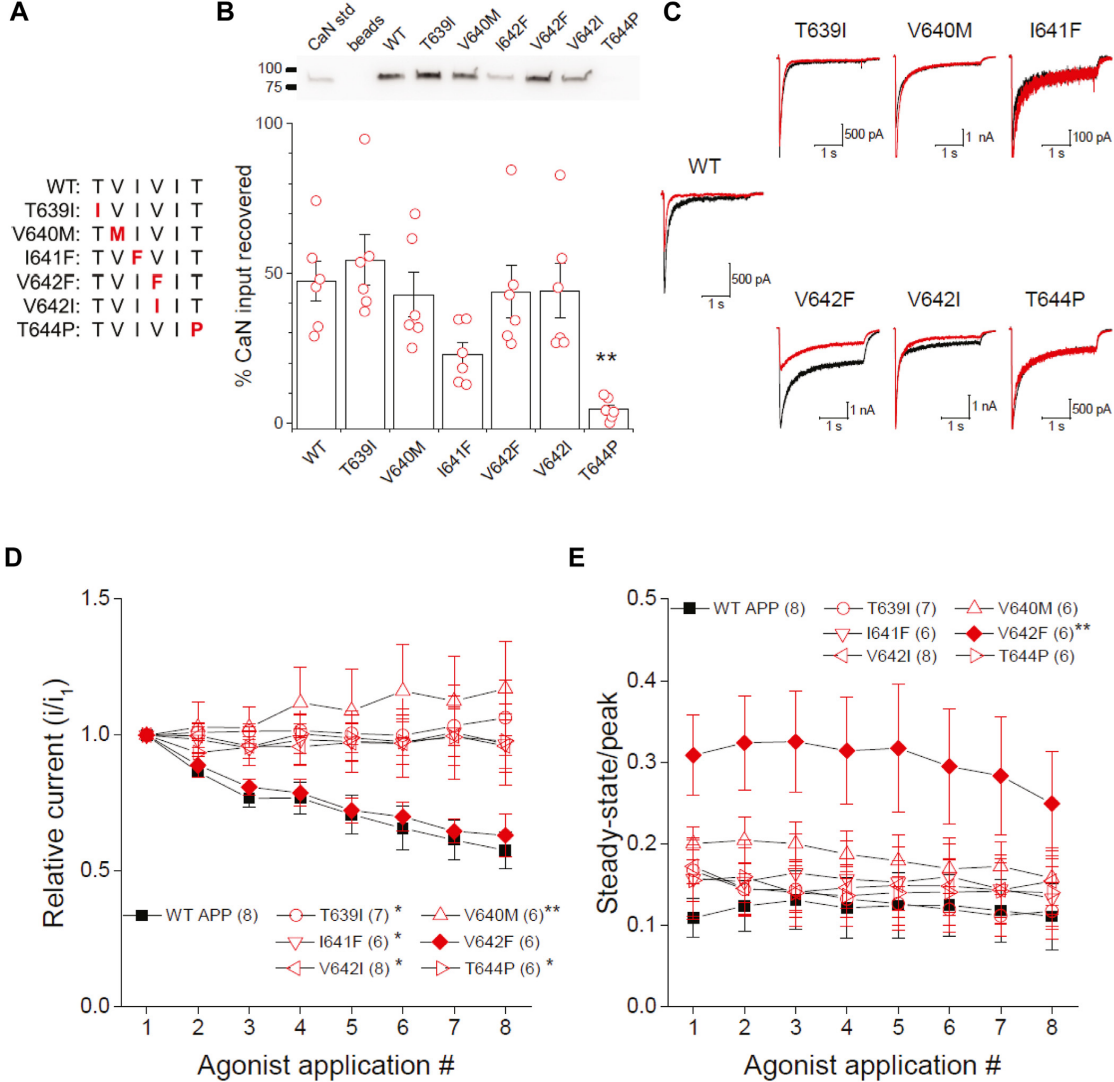
**FAD mutations impair the ability of APP to regulate recombinant NMDARs.***A*, sequence alignment of WT PxIxIT-like CaN-binding motif of APP with tested FAD mutants. The corresponding mutated residue is bolded and shown in *red*. *B*, *in vitro* interaction of recombinant GST-CaN (200 ng) with biotinylated WT APP peptide or APP peptides incorporating the indicated FAD mutations. A representative blot is shown (*upper panel*). Summary bar graph from multiple experiments is shown (*lower panel*). Data are normalized to the signal from the CaN standard and adjusted by the fraction of input (40%) represented by the standard and expressed as mean ± s.e.m. Individual data points are overlaid and represent biological replicates. *C*, representative first (*black*) and last (*red*) NMDAR currents evoked by the application of 1 mM glutamate in the continuous presence of 100 μM glycine from HEK 293 cells expressing GluN1A/GluN2A receptor subunits and either APP-GFP or the indicated GFP-tagged FAD mutants APP. EGTA (0.2 mM) was included in the intracellular pipette solution. *D*, summary time course of the peak current response of each application normalized to the initial agonist application under conditions used in (*C*). *E*, summary time course of the extent of desensitization measured as the ss/p ratio for each agonist application under conditions used in (*C*). Data for (*D* and *E*) are represented as the mean ± s.e.m. The number of observations for each condition is indicated in parentheses and represents biological replicates. ∗*p* < 0.05; ∗∗*p* < 0.01 evaluated by one-way ANOVA followed by Dunnett’s *post hoc* using the WT peptide as the reference group (*B*: F_(6,35)_ = 5.955; *p* = 0.000230 ∗∗) and repeated measures ANOVA (*D*: F_(6,40)_ = 3.944; *p* = 0.003 ∗∗; *E*: F = 2.542; *p* = 0.035 ∗) followed by one-tailed Dunnett’s *post hoc* using APP WT as the reference group for comparison. Data for WT APP shown in panels (*C*–*E*) are from Figure 2. The representative traces for WT APP shown in panel (*C*) was from a different cell within the same dataset.

The APP CaN-binding domain is localized in a hotspot for FAD mutations (46). Currently, no proteins other than APP itself and the γ-secretase appear to bind within this region (26, 34, 47). While most mutations in this region elevate the Aβ42/Aβ40 ratio, they exhibit complex effects on γ-secretase cleavage and processing (46, 48). The ability of a subset of these mutations (Fig. 4*A*) to modify CaN interaction with APP was evaluated using the previously established *in vitro* assay. Of the mutations examined, only T644P had reliably reduced CaN binding relative to the APP (WT) peptide (Fig. 4*B*). While CaN binding to I641F was reduced substantially, this did not reach statistical significance (Fig. 4*B*). Other mutations (T639I, V640M, V642F, and V642I) exhibited similar overall CaN binding to APP (WT), yet most mutants within the interior positions of the motif (V640M, V642F, and V642I) exhibited notable variability in their capacity to bind CaN across multiple assays (Fig. 4*A*). This may be indicative of their ability to adopt different conformations with variable stability toward interaction with CaN, which may be beyond the resolution of this assay.

To discern the impact of these FAD mutations on CaN signaling within a cellular setting, they were incorporated into APP-GFP and co-expressed with GluN1A/GluN2A NMDARs in HEK 293 cells. Except for V642F, all APP FAD mutants examined exhibited an enhanced stability, across multiple agonists applications, of peak NMDAR current compared to the WT APP, yet desensitized to a similar extent as WT APP during each agonist application (Fig. 4, *B*–*D*). This effect on amplitude stability is commensurate with that observed in the presence of CsA or high BAPTA concentrations (Fig. 3*D*) and thus is consistent with the idea that these mutants disrupt APP-dependent CaN signaling to the NMDA receptor. The V642F FAD mutant, on the other hand, reduced the extent of NMDAR desensitization during individual agonist applications but still showed a decline in peak current following repeated agonist applications like WT APP (Fig. 4, *B*–*D*), consistent with the effects of CsA on desensitization in cells expressing APP (Fig. 3*C* vs. Fig. 2*A*). Collectively, these data further support the idea that APP regulates CaN access to substrates controlling two distinct processes that normally limit NMDAR activity and that FAD mutations in the APP CaN-binding domain can impair either of these regulatory pathways.

**Figure 5:**
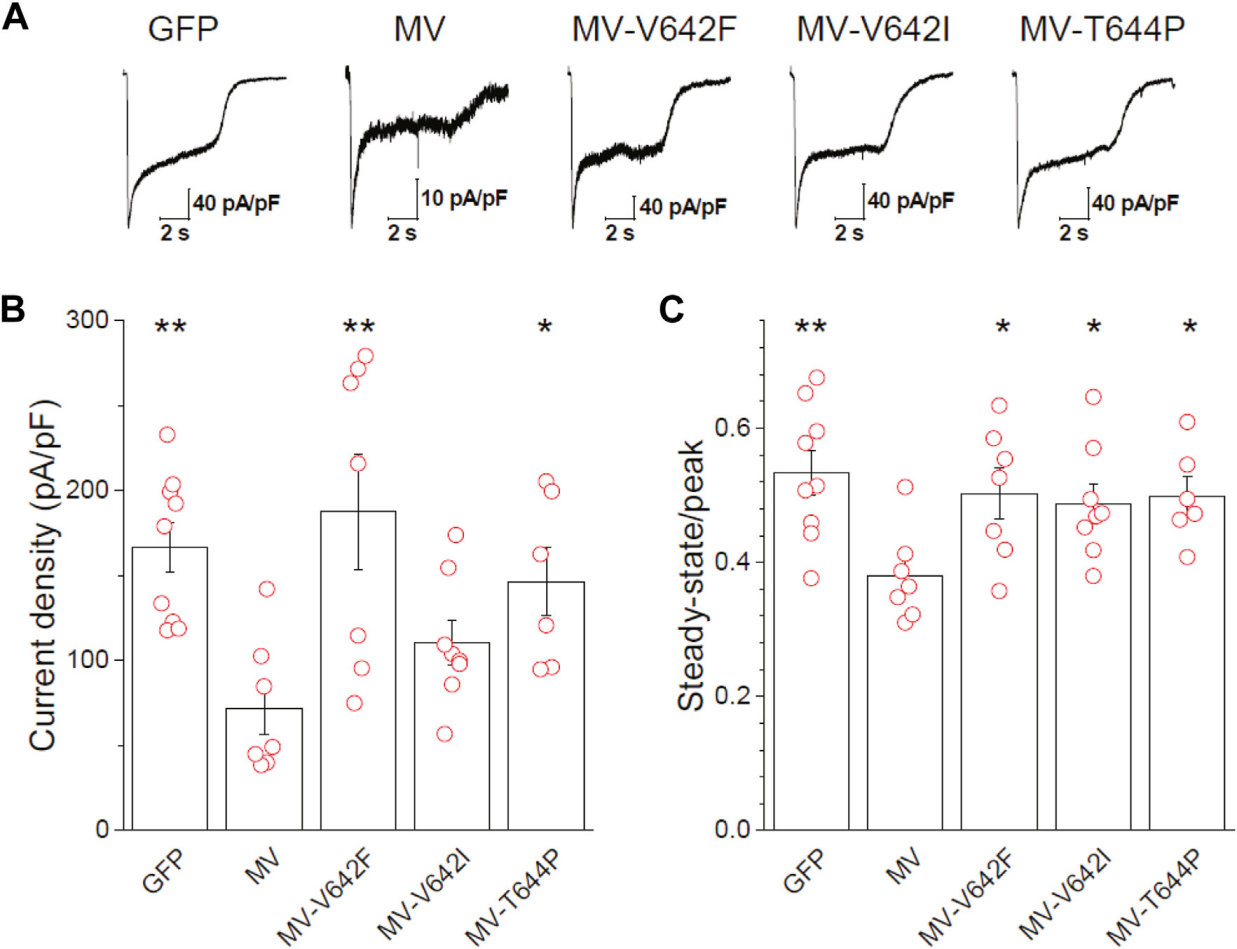
**FAD mutations impair the ability of APP to limit NMDAR activity in neurons.***A*, representative traces of NMDAR currents from cultured hippocampal neurons transfected with GFP or GFP-tagged APP or FAD mutants, which all have incorporated the M596V (MV) mutation that prevents processing of APP by the β-secretase. NMDAR currents were evoked by 5 s applications of 1 mM glutamate in the continued presence of 100 μM glycine. Traces have been scaled to their peak and currents are shown as current density. *B*, summary bar graph of the current density obtained from multiple experiments for the conditions described in (*A*). *C*, summary bar graph of the extent of desensitization measured as the steady-state/peak ratio for each agonist application under conditions used in (*A*). Overlaid data points (*B* and *C*) represent biological replicates. ∗*p* < 0.05; ∗∗*p* < 0.01 evaluated by ANOVA (*B*: F(_4,36_) = 5.209; *p* = 0.00239 ∗∗; *C*: F_(4,36)_ = 3.413; *p* = 0.0256 ∗) followed by one-tailed Dunnett’s *post hoc* using the MV group as the reference group for comparison.

Initial attempts to examine the impact of APP on native NMDARs *via* overexpression in cultured hippocampal neurons were unsuccessful, as virtually all cells that could be recognized as expressing APP-GFP, in addition to many nearby cells, appeared severely deteriorated by 48 h post-transfection and unamenable to whole-cell recordings. Indeed, APP overexpression likely enhances the secretion of Aβ from transfected neurons (49, 50), which could have a negative impact on neuronal viability. While others have used early post-transfection time points to examine the impact of APP overexpression on synaptic function (49, 51), this was not feasible under the condition employed here, as APP-GFP expression was not easily discernible at 16 to 24 h post-transfection. At these early time points, APP-GFP expression was weak and diffuse, which may reflect processing of APP-GFP. To limit the confounds associated with APP processing, an M596V mutation was incorporated into APP-GFP and the V642F, V642I, and T644P C-terminal GFP constructs, referred to here as MV, MV-V642F, MV-V642I, and MV-T644P, respectively. The M596V mutation prevents cleavage of APP by the β-secretase, the rate-limiting step in the generation of Aβ (49, 52). When these constructs were cotransfected with mCherry, into cultured hippocampal neurons, transfected neurons were identifiable by mCherry epifluorescence and GFP epifluorescence readily confirmed expression of the various APP constructs at 48 h post-transfection. Despite this improvement, the poor health of these neurons typically limited recordings to a single glutamate application. MV-transfected neurons had current densities that were less than 50% of GFP controls (Fig. 5, *A* and *B*) and desensitized to a much greater extent (Fig. 5, *A* and *C*). As CaN limits the amplitude and enhances desensitization of NMDAR currents (3, 5, 15, 16), both effects are consistent with the idea that APP limits NMDAR activity *via* CaN. NMDAR current densities were elevated in the presence of the MV-V642F and MV-T644P constructs, and the extent of NMDAR desensitization was reduced relative to that exhibited by the MV construct (Fig. 5, *A*–*C*). While the MV-V642I construct exhibited a noticeably elevated NMDAR current density compared to the MV construct, this did not reach statistical significance. Nonetheless, like the other FAD mutants, the MV-V642I reduced the extent of NMDAR desensitization. Thus, despite a clear distinction among the FAD mutants in terms of their impact on NMDAR amplitude stability and kinetics when examined in HEK 293 cells, this distinction was not evident when examined in native system. Indeed, each mutant tested in the native system tended to show an increase in amplitude and uniformly exhibited reduced extent of desensitization compared to the MV construct. Whether this arises from the use of the M596V mutation in neurons, which could limit variability due to differential processing of the mutants that may be operant in HEK cells is unclear. Regardless, in both systems, desensitization processes that control NMDAR amplitude and kinetics are enhanced by the presence of APP and disrupted by FAD mutations in the APP CaN-binding domain. While these data may superficially suggest that the FAD mutants rescue the MV phenotype to resemble the GFP controls, it is important to emphasize that cells expressing the FAD mutants were very fragile and thus unlike GFP controls. It is likely that the loss of feedback CaN signaling to NMDARs in these mutants contributes their declining health.

## Discussion

Although APP is absent from NMDAR receptor complexes using a mass spectrometry–based approach (53), CaN and APP have been variably detected along with NMDARs in some surveys of the postsynaptic density (54). While CaN and AKAP150 have been found in the NMDAR complex (53), the ability of AKAP150 to recruit CaN to NMDARs remains uncertain (55), leaving open the identification of proteins that may enable CaN recruitment to NMDARs despite extensive recognition of the ability of these receptors to be modulated by CaN (3, 5, 11, 12, 15, 16, 17). Likewise, NMDARs and CaN have not been reported within APP complexes evaluated by mass spectrometry (56). Whether the absence of these proteins reflects a bias in these studies towards more abundant and/or more persistent interactions and/or is reflective of the developmental stage, tissue/cell type, and/or the conditions under which these complexes were isolated and evaluated is not clear. Nonetheless, targeted co-immunoprecipitation studies from native and heterologous expression systems clearly indicate that APP interactions with either NMDARs (29, 30, 31, 32, 57) or CaN (Fig. 1) are resolvable. In fact, while this study was underway, a large-scale yeast two-hybrid screen reported that GluN1 and CaN each interact with APP (58), buttressing the idea, raised here, that APP may represent a key element connecting CaN to NMDARs. GluN1, APP, and CaN occupy overlapping territories within dendritic spines (59), thus lending further credence to the idea that these proteins may use this shared space to form a functional unit. While a ternary interaction between these proteins remains to be biochemically resolved, the data provided here and prior literature strongly supports its existence. Indeed, GluN1 (29) and CaN (Fig. 1) appear to bind to distinct regions of APP, which would be permissible for assembly into a ternary complex. Such a complex may be expected to recruit CaN close enough to the NMDAR, such that even strong Ca^2+^ buffering with BAPTA would not be able to curtail its regulatory action on the ss/p ratio but a CaN inhibitor would, as evident here (Fig. 3*D*). Thus, the combined biochemical, electrophysiological, molecular, and pharmacological approaches used here strongly support the idea that APP functionally links CaN to NMDARs.

Our understanding of the endogenous physiological functions of APP has been relatively elusive despite its recognition as the source of the amyloid peptides that deposit as plaques, representing a pathophysiological hallmark of AD. Numerous difficulties in understanding endogenous APP function within native settings have been noted and include segregating the effects of APP from its processing products, redundancy and/or interaction with other APP family members, overexpression and/or compensatory artifacts, and contributions from developmental alterations (26, 60, 61). In addition to these factors, one must also consider the prevailing activity state and its interplay within neuronal networks (38, 62), as well as any ensuing wave of damage to neighboring neurons associated with models linked with elevated Aβ (50, 63). Spurred by initial observations that APP and CaN interact in native tissue, the reductionist approach used for subsequent experiments here limits many of these confounds and consequently allows clearer resolution of the capacity of APP and its related family members to regulate NMDARs. Indeed, using this approach, the studies here define a new functional property of APP, which may be relevant to multiple NMDAR-mediated phenomena tied to dysfunctional CaN signaling in various AD models as well as for pharmacotherapeutic strategies for AD. While experiments in native systems confirmed that the key aspects of the regulation of NMDARs by APP-anchored CaN discerned in recombinant systems also apply to native systems, interpretation of overexpression experiments in neurons must be done with caution as APP and NMDARs appear to interact only at a subset of synapses (41). Indeed, it is unclear whether the various overexpressed APP constructs replaced endogenous APP at these sites, or enabled regulation at new sites, or a combination of both. Nonetheless, the use of whole-cell agonist–evoked currents provided a means to probe the entire population of NMDARs and resolve the capacity of APP-anchored CaN to control native NMDARs receptors.

As shown here, APP targeting of CaN to NMDARs normally limits NMDAR activity by favoring desensitized states. The reduction in this process due to FAD-related mutations in the APP CaN-binding domain would be expected to expand the temporal window and dynamic range for NMDAR-mediated Ca^2+^ entry, which is intimately linked to the ability of NMDARs to induce multiple forms of synaptic plasticity (64). This would be expected to lead to shifts in the threshold for inducing various forms of synaptic plasticity, either impairing some or exaggerating others (64). Either of these situations might interfere with the acquisition of recent events and contribute to the earliest cognitive impairments associated with AD. While such metaplastic shifts in the threshold for synaptic plasticity have been observed in one AD model, these shifts occurred in the absence of any detectable change in basal NMDAR activity (65). However, as is common practice, NMDAR activity was monitored at positive potentials (65), where the influx of Ca^2+^ and the ensuing CaN activation would be minimized. It is possible that repetitive stimulation patterns could be more likely to reveal CaN-mediated changes in NMDAR activity (15, 16) and serve as a more appropriate reflection of the induction signal for synaptic plasticity. Thus, whether altered NMDAR activity contributes to shifts in the threshold for synaptic plasticity in various AD models requires further elaboration. In contrast to the reduction in NMDAR currents described here, others have reported either no change (49, 51) or an enhancement (30) of synaptic NMDAR currents in the presence of a comparable MV construct. Whether this reflects differences in the neuronal preparation, timing between transfection and recording, and/or recording conditions is not clear. Alternatively, it is possible that the regulation described here predominantly targets extrasynaptic NMDARs, which may be critical for Aβ-induced alterations in synaptic plasticity and neurotoxicity (66, 67).

A hyperexcitable state is evident in early AD and in mouse models (38, 62, 68), which frequently incorporate mutations within the APP CaN-binding domain. The enhancement of NMDAR activity, due to the presence of FAD-related mutations within the APP CaN-binding domain, could contribute to this state. Of note, memantine, an NMDAR antagonist, appears to favor Ca^2+^-dependent forms of NMDAR desensitization (69). As such, memantine may counteract the desensitization impairments and ensuing hyperexcitability associated with the CaN-binding domain mutations in APP. This action may be therapeutically beneficial particularly at the earliest stages of FAD and contribute to its preclinical benefit evident in many mouse models, including those that incorporate mutations in the APP CaN-binding domain (40, 70).

Whether late-onset forms of AD involve alterations in NMDAR desensitization remains unclear. Yet, Aβ peptides, which accumulate in AD, can increase whole-cell NMDAR currents by reducing the extent of desensitization (71). This Aβ-induced reduction in desensitization effectively phenocopies the effects of the FAD mutants shown here, suggesting NMDAR desensitization may be a common parameter shared between early-onset FAD and the more common sporadic forms of AD. The effect of Aβ on NMDAR desensitization has been linked, in part, to its glycine sensitivity (71). As CaN controls the conversion between glycine-dependent and glycine-independent modes of desensitization (5), it is possible that CaN plays a role in Aβ-driven shifts in the sensitivity of desensitization to glycine. Because APP interacts with NMDARs *via* its glycine-binding GluN1 subunit (29), APP might represent a conduit by which CaN controls the glycine-sensitivity of NMDAR desensitization.

The ramifications of the overlap of the CaN-binding domain within the γ-secretase–processing region of APP could be manifold. Indeed, activity-dependent processing of APP has been linked to NMDARs (30, 41, 49, 72). Thus, one can envision a scenario in which the occupancy of CaN at this site normally limits processing. NMDAR activity and the ensuing Ca^2+^ entry could then drive CaN activation and displacement from this site, thereby allowing γ-secretase access to this site for eventual Aβ generation. It is plausible that a related scenario could contribute to the enhanced synaptotoxic effects of longer Aβ species due to their increased propensity to self-aggregate (73). In this situation, the binding of longer Aβ peptides to its cognate region on APP could sterically hinder CaN targeting by APP to a greater extent that shorter peptides and thus favor γ-secretase access to this region of APP. Displacement of CaN from APP, as well as proteolytic destruction of the CaN-binding domain by the γ-secretase complex, would be expected to acutely enhance NMDAR activity. Indeed, acute enhancement of NMDAR activity *via* various Aβ species has been reported (66, 71, 74, 75, 76). This increase in NMDAR activity might then be able to broadcast its Ca^2+^ signal to downstream CaN targets more effectively, eventually culminating in the downregulation of either NMDA and/or AMPA receptors (77, 78, 79, 80, 81, 82). In support of this latter idea, Aβ relies on the presence of APP to mediate its effects on synaptic plasticity (63, 83, 84) and Aβ induces exaggerated CaN signaling (77, 78, 79, 80, 81, 85, 86, 87, 88), which is often linked to altered NMDAR-mediated signaling (78, 80, 81). The abatement of these downstream effects may contribute to the beneficial cognitive effects of CaN inhibitors in rodent AD models (89, 90, 91) and humans (92, 93).

The reliance of the APP family of proteins on a modified PxIxIT-like motif to target CaN for NMDAR regulation suggests that the first position of the motif is more tolerable to amino acids substitution than originally envisioned (19). For example, APP and the T639I mutant bind CaN to a similar extent as the PVIVIT peptide, despite their degeneracy from the motif at this position. Thus, the repertoire of CaN-binding proteins within the proteome may be more extensive than currently appreciated (18, 19). The relative affinity of peptides based on APP-family members to associate with CaN appears to correspond well with the ability of each member to modulate NMDARs and suggests that the PxIxIT-like motif present in APP family members suffices for CaN binding and the ensuing CaN-mediated regulation of NMDARs. However, it is notable that all APP FAD mutants disrupted CaN signaling despite, in most cases, negligible alterations to its apparent affinity for CaN. It is possible that additional sequences within APP contribute to the overall ability to recruit CaN for effective NMDAR regulation. Nonetheless, even modest shifts, in either direction, in the affinity of anchoring proteins for CaN may hinder CaN signaling to relevant substrates (27). Yet these mutations have an outsized effect in the context of the intact protein within a cellular environment suggesting that the residues within this PxIxIT-like motif play a critical role in orienting CaN activity to NMDARs. An altered regulatory landscape for CaN is exemplified by the V642 site mutants, which discriminate between two forms of NMDAR regulation, despite a superficially similar ability to bind CaN. This sensitivity to the identity of residues within the APP CaN-binding domain suggests that the WT APP sequence has been evolutionarily optimized to ensure tight control of NMDAR activity by CaN. The fact that APLP2 exhibits similar CaN-binding and ability to regulate NMDARs regulation as APP may underscore this idea, as well as contribute to the overlapping functions attributed to these family members (26). At present, the target protein(s) and corresponding phosphorylation sites controlled by APP-anchored CaN underlying the regulation of NMDAR activity remain unclear, albeit phosphorylation sites in NMDAR subunits (3, 94) and APP (95) are chief candidates. Identification of these sites and the relevant opposing signaling pathways may enable the development of new agents and/or strategies, which can target these pathways for the treatment of the earliest stages of Alzheimer’s disease.

## Experimental procedures

### Plasmids and site-directed mutagenesis

Plasmids encoding GluN1A (GenBank U08261.1), GluN2A (GenBank D13211.1), and GluN2B (GenBank U11419.1) in pcDNA1/AMP were previously described (Krupp *et al.* 1996) and obtained from Gary Westbrook. The CaNA subunit in pCDNA3.1(+)MycHis(A) was previously described (20) and obtained from Mark Dell’Acqua. APP(695) and Flag-APP(695) in pCAX were gifts from Dennis Selkoe & Tracy Young-Pearse (Addgene plasmid # 30137 and 30141, respectively). APP in pEGFP(N1) was a gift from Zita Balklava & Thomas Wassmer (Addgene plasmid # 69924). APLP1 (GenBank BC012889.1) in pCMV-Sport6 and APLP2 (GenBank BC000373) in pOTB7 were purchased from Dharmacon. An EcoR1/Xba1 fragment from APLP2 was ligated into the corresponding site in pCMV-Sport6. A Flag-tag was incorporated immediately after the signal sequence of APLP1 and APLP2 in pCMV-Sport6 using the QuikChange lightning mutagenesis kit (Agilent). PCR fragments encompassing the coding regions of APLP1 and APLP2 were ligated into SacI/SmaI and SacII/SmaI sites into GFP-N1 to generate APLP1-GFP and APLP2-GFP, respectively. The overlap-extension mutagenesis method was used to introduce all FAD mutants into APP-GFP. The QuikChange method was used to introduce the M596V mutant into APP-GFP (designated as MV). EcoRI/SalI fragments from the following FAD mutants: V642F-GFP, V642I-GFP, and T644P-GFP were ligated into the corresponding sites in MV to generate the MV-V642F, MV-V642I, and MV-T644P, respectively, which all retained the C-terminal GFP tag. In all cases, DNA sequencing was performed by either the UTHSC Molecular Resource Center or Azenta to verify the correct incorporation of the tags and mutations.

### Antibodies, recombinant protein, and peptides

Recombinant rabbit monoclonal to APP (clone Y188; Abcam cat# ab32136; 1:5000 dilution), rabbit polyclonal to APLP1 (Proteintech cat # 12305-2-AP; 1:1000 dilution), and rabbit polyclonal to APLP2 (Proteintech cat # 15041-1-A; 1:500 dilution) were used to detect native APP family members on western blots or recombinant GFP-tagged APP family members as validation (see Fig. S1). A horseradish peroxidase (HRP)-conjugated goat anti-rabbit antibody (Sigma-Aldrich Cat# 12-348; 1:10,000 dilution) was used as secondary antibody for these western blots, while an HRP-conjugated mouse monoclonal antibody to GFP (Invitrogen cat # MA 515256HRP; 1:1000 dilution) was used to independently verify the expression of GFP or GFP-tagged APP family members. A mouse monoclonal antibody CaN (BD Biosciences Cat# 556350; 1:1000 dilution) was used for the detection of native and recombinant CaN on western blots and for co-immunoprecipitation and peptide-based interaction assays experiments, while nonspecific mouse IgG (Thermo Fisher Scientific Cat# 02-6502) was used as a control. An HRP-conjugated goat anti-mouse antibody (Sigma-Aldrich Cat# 12-349; 1:10,000 dilution) or protein was used as secondary antibody for these western blots, while HRP-conjugated protein G (Sigma-Aldrich cat # 18-161; 1:5000 dilution) was used for the secondary detection of CaN in co-immunoprecipitation experiments. A mouse monoclonal anti-Flag antibody (Sigma-Aldrich Cat# F1804) was used for co-immunoprecipitations of recombinant APP family members, while an HRP-conjugated version of this antibody (Sigma-Aldrich Cat# A8592; 1:1000) was used for the detection on western blots. Peptide/CaN interaction assays used recombinant GST-tagged CaN A subunit (Abcam cat # ab128554).

The N-terminally biotinylated AKAP79(330-357) and Ht31peptides were synthesized by Biomolecules Midwest and used as positive and negative controls for CaN interaction as previously described (20). PVIVIT, APP family members, and corresponding APP FAD mutants were synthesized as N-terminally biotinylated peptides (Lifetein) and had the following sequences: PVIVIT-MAGPHPVIVITGPHEE; APP(17-mer)-GVVIATVIVITLVMLKK; APP-MAGPHTVIVITGPHEE; APLP1-MAGPHSLIVLSGPHEE; APLP2-MAGPHTVIVISGPHEE; T639I-MAGPHIVIVITGPHEE; V640M-MAGPHTMIVITGPHEE; I641F-MAGPHTVFVITGPHEE; V642F-MAGPHTVIFITGPHEE; V642I-MAGPHTVIIITGPHEE; T644P-MAGPHTVIVIPGPHEE.

### Cell culture and transfections

HEK 293 cells (ATCC Cat# CRL-1573) were obtained at passage 37 and used for a maximum of six passages. Cell cultures were maintained in Dulbecco’s modified Eagle’s medium (DMEM) with 10% fetal bovine serum (Life Technologies, Inc.) and penicillin/streptomycin (P/S). For electrophysiology, cells were plated at low density (∼50,000 cells/ml) on 15-mm round glass coverslips in 12-well plates. Cells were transfected using the Ca^2+^ phosphate method with 1 μg of each construct per coverslip. For control cells, 0.3 μg of pEGFP (GenBank U55762.1) was included as a transfection marker. Epifluorescence was used to confirm the expression of the corresponding APP family members and APP FAD mutants. For biochemistry, HEK 293 cells were plated at ∼50% confluence on 10 cm dishes. HEK 293 cells were transfected by the Ca^2+^ phosphate method. For these experiments, 4 μg of each construct was used, with 1 μg of mCherry (GenBank AY678264.1) used as a transfection marker. Experiments were performed 24 h after transfection.

Neuronal cultures were prepared by removing the hippocampi from neonatal (1–2 days old) Sprague-Daley rats of either sex following Institutional Animal Care and Use Committee approved protocols. Tissue was dissociated by papain (20 U/ml; Sigma) treatment and trituration through pasteur pipettes and suspended in media consisting of the following: DMEM (Life Technologies, Inc. Cat# 11965-092) with 10% FBS and P/S. Cells were plated at a density of 125,000 cell/ml on poly-D-lysine and collagen-coated 15-mm round glass coverslips in 12-well plates. Cells were washed with this media 24 h after plating and then subsequently exchanged with DMEM (Life Technologies, Inc Cat# 10313-021) supplemented with B-27 plus (Life Technologies), 10 mM Hepes, 0.5 mM GlutaMax, and P/S after 24 h later. One-half of the media was refreshed every 3 days. Media was supplemented with 5-Fluorouracil and uridine to prevent glial cell proliferation ∼6 days after plating. Cells were transfected at 8 to 12 days *in vitro* using Lipofectamine 2000. Prior to transfection, 1 ml of fresh media was added to each well and 1 ml was subsequently removed and reserved as post-transfection media. Transfection reactions were prepared by adding 4 μl to 100 μl OptiMEM (Life Technologies), mixing, and then allowing it to sit for 5 min. DNA (0.8 μg mCherry, and 0.8 μg of GFP or MV-GFP, or FAD mutants of MV-GFP) was added to the reaction and mixed upon which it sat for 20 min. The transfection reactions were then added to the cells, which were then placed in an incubator for 30 min. Cells were then washed once with PBS and then replaced with the media that was initially removed.

### Co-immunoprecipitations and western blotting

Sprague-Dawley rats (Harlan) 24 to 28 days old, of either sex, were sacrificed according to protocols approved by the University of Tennessee Health Science Center Institutional Animal Care and Use Committee. Brains were rapidly removed and frozen in liquid nitrogen. Frozen brains were pulverized and then Dounce homogenized in ice-cold lysis buffer (150 mM NaCl, 10 mM Hepes, 5 mM EDTA, pH 7.4) and protease inhibitors (Sigma cat #P8340), pH 7.4) with 1% CHAPSO added. The lysate was clarified by centrifugation at 14,000 g for 10 min. Protein concentration for the resulting supernatant was determined by the Bradford assay (Bio-Rad), and the extract was subsequently diluted to 2 mg/ml. 0.5% CHAPSO was added to the lysis buffer to generate the IP buffer used for co-immunoprecipitations. Protein G beads (20 μl; Invitrogen cat# 10004D) were washed in IP buffer and incubated with 4 μg of either anti-CaN or control mouse IgG antibodies in 200 μl IP buffer for 30 min and then washed 3×. Beads were then incubated with 400 μg (200 μl) of brain lysate overnight and then subsequently washed 3× with IP buffer (5 min; 400 μl each) and then eluted with 40 μl 2× Laemmli buffer. Ten micrograms (2.5%) of the brain extract was used for input lanes representing. Samples were boiled for 5 min and subsequently resolved on 4 to 20% SDS polyacrylamide gels (BioRad) and transferred to nitrocellulose and stained with Ponceau S solution (Sigma) to verify successful transfer. Membranes were blocked with 3% nonfat dry milk in tris buffered saline containing 0.05% Tween-20 for 1 h. Following washing, blots were probed with anti-CaN antibodies for overnight and then washed and probed with protein G-HRP for 1 h. Following washing, immunoblots were visualized by enhanced chemiluminescence (Thermo Fisher Scientific). Data were digitally acquired and quantified using a Bio-Rad XRS chemiluminescence documentation system and Quantity One software. Gels were stripped with a glycine-based buffer (0.2 M glycine, 0.1% SDS, 1% Tween-20; pH 2.2), blocked, and reprobed for the indicated APP family members with appropriate secondary antibodies as above. For co-immunoprecipitation experiments from transfected HEK 293 cells, each 10 cm plate was lysed in 400 μl IP/lysis buffer containing 1% CHAPSO and clarified as above. Two hundred microliters of the resulting extract was incubated with streptavidin beads that had been coupled with 4 μg of either anti-Flag or nonspecific mouse IgG as above. Ten microliters (5%) of input was loaded as the extract lane on gels. Blots were probed with the anti-CaN antibody followed by protein G-HRP for secondary detection. Following digital acquisition of the resulting image, blots were stripped and reprobed with an HRP-conjugated anti-Flag antibody and visualized by chemiluminescence. Data shown are representative of at least three experiments.

### Peptide/CaN interaction assays

Either PBS with fresh 0.1% Tween-20 and 0.1% bovine serum albumin added or a modified IP buffer (150 mM NaCl, 10 mM Hepes; 5 mM EDTA; pH 7.4) with fresh 0.5% CHAPSO and 0.1% bovine serum albumin was used for these assays. Assays performed using either buffer yielded comparable results and thus the data were aggregated. Streptavidin-coated dynabeads (20 μl; Invitrogen cat # 65601) were washed and then incubated with 10 μg biotinylated peptides for 15 min at 4 °C in 200 μl of buffer. Beads were washed rapidly (400 μl; 3×) to remove unbound peptide and then incubated with 200 ng human recombinant GST-CaN in 200 μl buffer for 30 min at 4 °C. Following three washes (400 μl; 5 min each), complexes were eluted in 40 μl 2× Laemmli buffer, and samples were separated by SDS-PAGE and analyzed by Western blotting as above. Blots were probed with the mouse monoclonal anti-CaN antibody followed by anti-mouse HRP for secondary detection and visualized by enhanced chemiluminescence and digitally acquired as above. For each blot, the intensity of each band was normalized to that of from the GST-CaN standard (80 ng). As this standard represented 40% of the 200 ng input, the ratio was multiplied by 40 to achieve the percent of CaN recovered.

### Electrophysiology

Twenty-four hours after transfection, HEK 293 cells were visually selected for recording by GFP epifluorescence. While neuronal recordings were performed 48 h post-transfection, whole-cell recordings of NMDA receptor currents were obtained with a Multiclamp 700A amplifier (Molecular Devices) and digitized through a Digidata 1332A using Clampex 10.3 software. Patch pipettes (2–5 MΩ for HEK 293 cells; 3–6 MΩ for neurons) contained (in mM) the following: 140 Cs methanesulfonate, 10 Hepes, five ATP (Na salt), 5 MgCl2, and 0.2 EGTA + 0.01 CaCl2 or 10 BAPTA + 0.2 CaCl2 (pH 7.4). QX-314 (2 mM) was included in the pipette solution for neuronal recordings to ensure the block of voltage-gated Na^+^ currents. The extracellular solution contained (in mM) 150 NaCl, 5 KCl, 1.8 CaCl2, 10 Hepes, 11.1 glucose, 0.1 glycine (pH 7.4) and flowed at a rate of 1 to 2 ml/min. For neuronal recordings, this solution was supplemented with tetrodotoxin (0.3 μM), NBQX (10 μM), and picrotoxin (100 μM), to block voltage-gated Na^+^ channels, AMPA receptors, and GABA receptors, respectively. HEK cells were lifted from the coverslip after establishment of the whole-cell configuration, to speed solution exchange, and placed ∼20 μm from the mouth of a series of flow pipes, which were controlled by solenoid valves and moved into position by a piezoelectric bimorph. With this configuration, solution exchanges were accomplished within 10 to 12 ms as assessed by the rise time (20–80%) of whole-cell agonist-evoked current responses. Flow pipes were lowered to within ∼50 μm of the surface of the coverslip and placed ∼100 to 150 μm from the soma of the cell. Currents were digitized at 2 kHz and filtered at 1 kHz. Series resistance (90–95% HEK cells; 60–70% neurons) and whole-cell capacitance compensation were employed, and input resistance was routinely monitored by 5 mV hyperpolarizing jumps between agonist applications. Cells showing sudden shifts in these parameters were discarded. Experiments were performed at a holding potential of −60 mV at 20 °C. Currents were elicited by a 3 s (5 s for neurons) application of glutamate (1 mM) in the extracellular recording solution. Desensitization was quantified as the ratio of average current remaining in the last 50 ms of the agonist application to the peak current using Clampfit 10.3 software. For HEK cells, only cells which lasted at least eight agonist applications were included for analysis. For neurons, only the initial agonist application was used. Current density was calculated by dividing the peak current by the compensated whole-cell capacitance.

### Statistical analysis

All quantified data are presented as mean ± SEM. Sample numbers are indicated in the figure and in the Supporting Information. Statistical analysis was performed using SPSS software. One-way ANOVA with Tukey’s *post hoc* was used to evaluate the binding of CaN to PVIVIT and APP family members and the indicated control peptides in Figure 1. One-way repeated measures ANOVA test was used to evaluate differences between the various transfection groups for their peak amplitude of NMDAR current or ss/p ratio across multiple agonist applications. The s.e.m for all groups was 0 at this time point because amplitude data were normalized to the initial agonist application. As such, amplitude was evaluated from agonist applications 2 to 8. Steady-state/peak ratios were evaluated across all agonist applications. For Figure 2, Dunnett’s *post hoc* analysis was performed using GluN1A/GluN2A + GFP as the control group. As only two groups were compared in Figure 3, the omnibus F statistic sufficed to determine significance. For Figure 4, One-way ANOVA followed by Dunnet’s (two-tailed) *post hoc* was used to compare the binding of FAD mutant peptide to the WT APP peptide. For the electrophysiological experiments, WT APP was used as the control group by which FAD mutants were compared against using ANOVA followed by a one-tailed Dunnet’s *post hoc*. A one-tail test was considered appropriate for this test as results from Figure 3 suggested that if FAD mutations led to an impairment in CaN signaling, they would manifest as an increase in either NMDAR stability or the ss/p ratio. For Figure 5, a one-way ANOVA was followed by a one-tailed Dunnet’s *post hoc* using the MV group as the control group by which other groups were compared against. A one-tailed test was considered appropriate as results from earlier figures suggested that APP (or MV) expression would diminish either the amplitude of ss/p ratio compared to GFP and that FAD mutants would most likely act in the opposite direction. Statistical significance is indicated with ∗ or ∗∗ for *p* values below 0.05 or 0.01, respectively, while n.s. is used to designate nonsignificant results. Significance was defined as *p* < 0.05. All data and exact *p* values are reported in the Supporting Information.

## Data availability

All data needed to evaluate the conclusions in the paper are present in the paper and/or the Supporting Information. Plasmids generated in this study are available upon request.

## Supporting information

This article contains supporting information.

## Conflict of interest

The author declares that he has no conflicts of interest with the contents of this article.
