# Supplementary material for: Familial Alzheimer’s disease mutations in amyloid precursor protein impair calcineurin signaling to NMDA receptors
Source: J Biol Chem. 2024 Dec 26;301(2):108147. doi: 10.1016/j.jbc.2024.108147 (PMC11910330; doi:10.1016/j.jbc.2024.108147)
Supplement: Supporting information [file mmc1.pdf]

Supporting Information for

Familial Alzheimer's disease mutations in amyloid precursor protein impair calcineurin signaling to NMDA receptors

Steven J. Tavalin

*Corresponding author. Email: stavalin@uthsc.edu

This PDF file includes:

Figures. S1 to S2

Other Supplementary Materials for this manuscript include the following:

Data S1

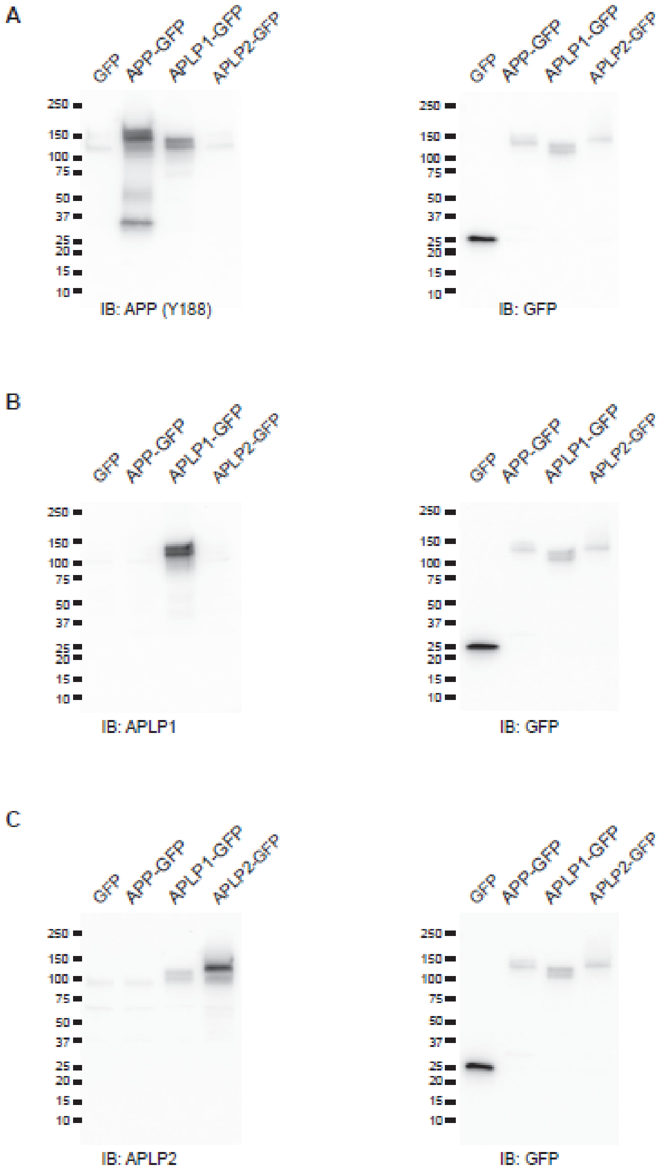

Figure S1: Validation of antibodies used to detect APP family members in brain extracts. HEK 293 cells were transfected with GFP, or GFP-tagged APP family members. The resulting extracts were separated by SDS-PAGE, transferred to nitrocellulose, and immunoblotted with antibodies to **(A)** APP, **(B)** APLP1, **(C)** APLP2 (left panels). Blots were stripped and subsequently immunoblotted with an antibody to GFP (right panels). The antibodies to APP and APLP2 preferentially recognize their intended targets, while the APLP1 appears to be specific for APLP1. Despite some recognition of APLP1 by the APP and APLP2 antibodies, APLP1 can be distinguished from APP and APLP2 based on its migration on the gel.

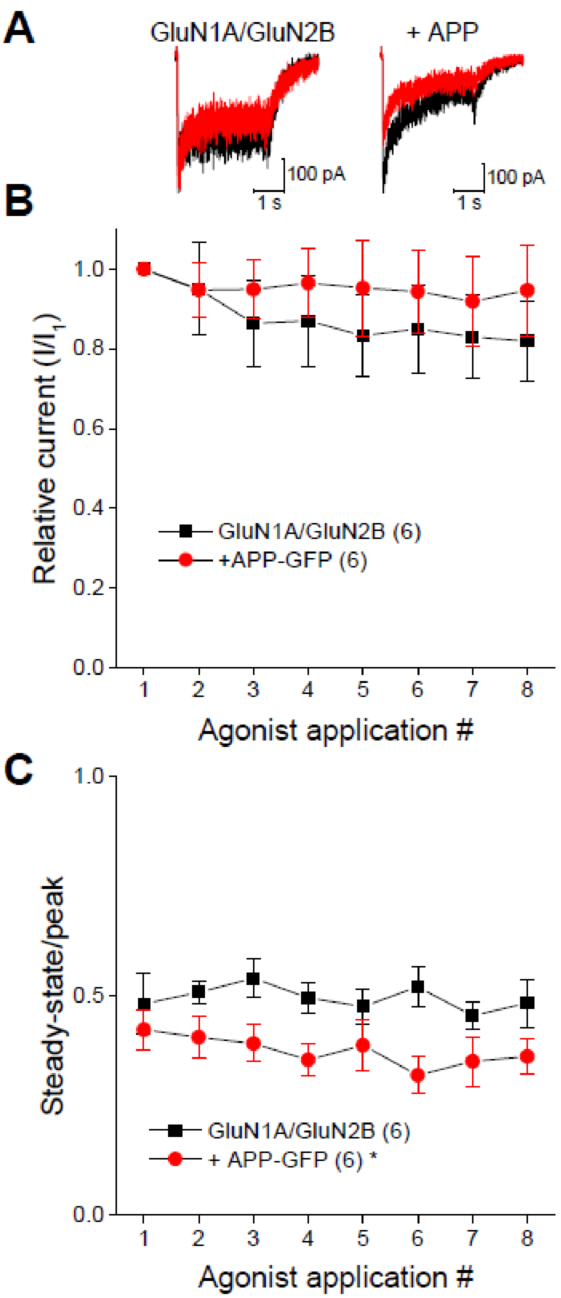

Figure S2: APP co-expression enhances desensitization of GluN1A/GluN2B NMDARs. (A) Representative first (black) and last (red) NMDAR currents evoked by application of 1 mM glutamate in the continuous presence of 100 mM glycine from HEK 293 cells expressing GluN1A/GluN2B receptor subunits and either GFP (left) or APP-GFP (right). Low concentrations of EGTA (0.2 mM) were included in the intracellular pipette solution. **(B)** Summary time course of the peak current response of each application normalized to the initial agonist application **(C)** Summary time course of the extent of desensitization measured as the steady-state/peak ratio for each agonist application. Data for (B, C) are represented as the mean ± s.e.m. The number of observations for each condition is indicated in parentheses and represent biological replicates. * $p < 0.05$ evaluated by one-way repeated measures ANOVA (B: $F_{(1,10)} = 0.392$; $p = 0.545$ n.s.; C: $F_{(1,10)} = 4.997$; $p = 0.0498$ *)

Data S1 (Separate file): Supplementary data and statistical summary
